# Simulating a population genomics data set using FlowSim

**DOI:** 10.1186/1756-0500-7-68

**Published:** 2014-01-31

**Authors:** Ketil Malde

**Affiliations:** 1 Institute of Marine Research, Nordnesgaten 50, Bergen, Norway

**Keywords:** Simulation, Second-generation sequencing, Population genomics, Shotgun metagenomics, SNP

## Abstract

**Background:**

The field of population genetics use the genetic composition of populations to study the effects of ecological and evolutionary factors, including selection, genetic drift, mating structure, and migration. Until recently, these studies were usually based upon the analysis of relatively few (typically 10–20) DNA markers on samples from multiple populations. In contrast, high-throughput sequencing provides large amounts of data and consequently very high resolution genetic information. Recent technological developments are rapidly making this a cost-effective alternative. In addition, sequencing allows both the direct study of genomic differences between population, and the discovery of single nucleotide polymorphism marker that can be subsequently used in high-throughput genotyping. Much of the analysis in population genetics was developed before large scale sequencing became feasible. Methods often do not take into account the characteristics of the different sequencing technologies, and consequently, may not always be well suited to this kind of data.

**Results:**

Although the FlowSim suite of tools originally targeted simulation of *de novo* 454 genomics data, recent developments and enhancements makes it suitable also for simulating other kinds of data. We examine its application to population genomics, and provide examples and supplementary scripts and utilities to aid in this task.

**Conclusions:**

Simulation is an important tool to study and develop methods in many fields, and here we demonstrate how to simulate a high-throughput sequencing dataset for population genomics.

## Background

Simulation is an important tool for developing and experimenting with methods for analysis of sequencing data. Several simulators exist, usually targeting specific data types or analyses. For instance, MetaSim [1] targets metagenomic samples, and SimSeq (St. John, unpublished) and Wgsim [2] target Illumina sequences.

As implied by the name, FlowSim [3] was originally developed for simulation of *de novo* genomics data on the 454 platform. Since its inception, it has grown into a flexible suite of tools that can be applied to a number of different uses, and here we demonstrate how it can simulate a population genomics data set consisting of Illumina reads.

A sequencing dataset for population genomics typically consists of reads from pools of individuals from a species, where each pool is taken from a specific populations or subpopulation of interest. By identifying and quantifying variants in the different pools, one can calculate the degree of divergence and population structure between the populations. In turn, this information can be used to study evolution [4,5], quantitative traits [6], and also constitutes an important tool for estimating biological diversity.

## The FlowSim suite

**Figure 1:**
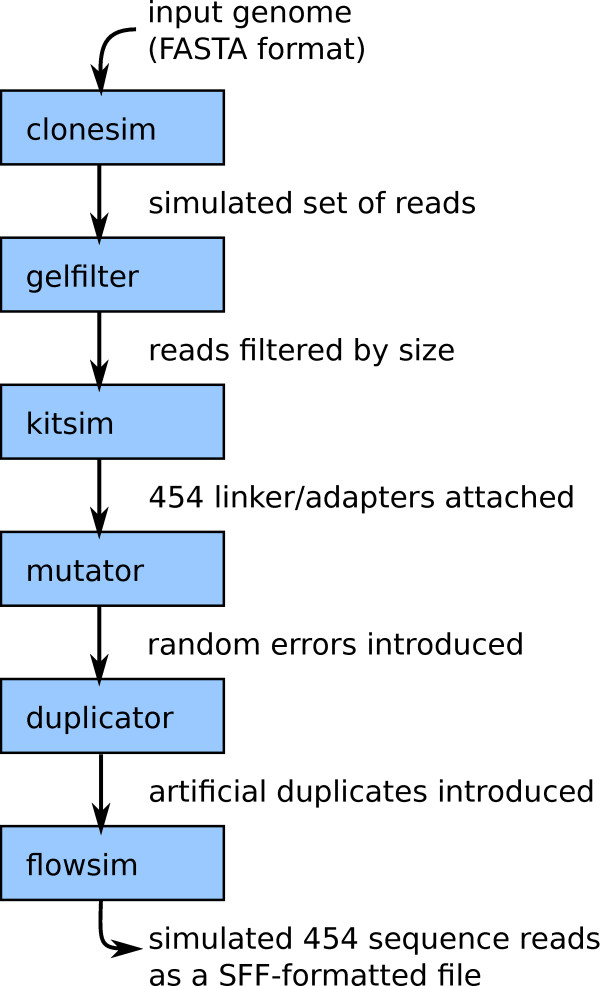
**Components of the FlowSim pipeline.** For a typical 454 sequencing simulation application, first the clonesim component takes a FASTA-formatted input genome and extracts random fragments representing the clones to be sequenced. The user can specify the statistical distribution to use for clone lengths. gelfilter then simulates filtering by sequence length (i.e. molecule size). kitsim simulates the attachment of 454-specific *adapters*, synthetic sequence fragments that are used in the sequencing process. mutator introduces random substitutions and indels into sequences. duplicator randomly increases the multiplicity of sequences, simulating the artificial duplicates that occur with most second generation technologies. Finally, flowsim simulates the 454 pyrosequencing process, and generates the final SFF file.

The current version of FlowSim (0.3.5) consists of several independent components, as illustrated in Figure 1. Each component is implemented as a separate tool, using FASTA-formatted sequences for input and output. (The exception is flowsim, which outputs the native SFF file format. FASTA-formatted sequence can be trivially extracted, e.g. using the flower[7] tool). This makes it easy for the user to construct a custom simulation pipeline tailored to his or her needs. Here, we will make use of clonesim to generate sets of reads, mutator to simulate genetic divergence in the form of SNPs as well as simulating sequencing errors in the simulated reads, and duplicator to introduce artificial duplicates.

## Methods and results

Under the current simulations, a population consists of a number of individuals with specific genetic variations. For simplicity, we will consider our populations as a sets of genome sequences, each similar to a reference genome, but differing in a set of locations with unique substitutions. We will refer to these genomes as the *haplotypes* of the population. Each haplotype (and thus its specific genomic variants) occurs with a specific frequency in the population as a whole.

Starting with a single haplotype (i.e., a reference genome or chromosome), we generate the new haplotypes by introducing random mutations. The mutations are identified, and noted separately. The resulting haplotypes are then concatenated in desired multiplicities into a combined genome representing each population, and sets of simulated reads are generated by selecting fragments randomly from the the population genomes. Finally, to simulate sequencing errors, artifacts [8], and the occurrence of rare variants [9], the reads have additional variations introduced. Also, a random selection of reads are output multiple times in order to simulate the occurrence of artificial duplicates [10,11].

## Implementation

We will presume that our reference genome exists in a file called genome.fasta. First the set of haplotypes are generated by using mutator to randomly introduce on average five mutations per kilobase, using the option -s 0.005. To simplify analysis, we do not introduce indels (-i 0). The following script implements the analysis. 

Although here we generate intermediate files, each step can also read from standard input and write to standard output. Thus, intermediate files can be omitted using UNIX pipes.

**Figure 2:**
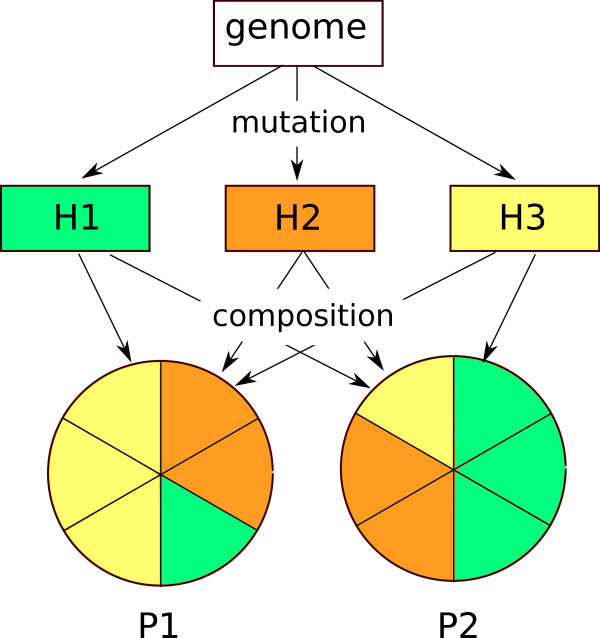
**Generating population genomes from haplotypes.** Three different haplotypes (labeled H1, H2, and H3) are generated from the reference genome by applying random mutations. The haplotypes are then concatenated in appropriate multiplicities so that mutations specific to each haplotype will occur with known frequencies in the population genomes (labeled P1 and P2).

The next step simply concatenates the haplotypes in different proportions to construct the population genomes, p1.fasta and p2.fasta. Here, we combined the three haplotypes *H*_1_, *H*_2_ and *H*_3_ in proportions of 1:2:3 in population *P*_1_, and 3:2:1 in population *P*_2_, as shown in Figure 2. As a result, an allele present in *H*_1_ (i.e., H1.fasta) will have a minor allele frequency of 0.167 in population *P*_1_, and 0.5 in *P*_2_, giving it an *a prioriF*_*ST*_ of 0.125, while variant alleles in *H*_2_ will occur with an equal minor allele frequency (of 0.333) in either population, resulting in an *F*_*st*_ of 0.

In step three, we can use clonesim to generate reads by extracting 20 M (-c 2000000) random fragments of exactly 100 bp length (using the -l option to set the length distribution to Uniform 100 100). The generated reads are exact copies of fragments of the reference genome, and in order to simulate sequencing errors and rare variants, in step four we again apply mutator, this time allowing indels as well as substitutions. Finally, we randomly duplicate some of the reads, using the duplicator tool.

## Additional analysis

FlowSim provides the basic building blocks for simulating the sequencing process, but analysis often depends on additional information, and sometimes requires intermediate steps to adapt the data.

A natural step in the analysis of sequence reads, simulated or otherwise, is to map them to a reference genome. This is also useful to verify that the data exhibits the expected properties, like coverage distribution or error rates. The simulation here produces FASTA sequences, but most short read mapping software accept FASTQ as input. Converting from FASTA to FASTQ is a simple task, here a small tool (called fasta2fastq) was written to perform this conversion.

To separate the haplotype variants from simulated sequencing errors, another small tool (snplist) were written to generate the list of variants per haplotype. This compares each haplotype with the reference genome, and outputs a list of the variant positions with reference and alternative allele. To simplify this process, it is conveneint to add the variants identification to e.g. the output from VCFtools [12] or similar variant callers, the following script can be used for this purpose: 

## Discussion and conclusion

As FlowSim is primarily targeted at accurate simulation of 454 sequencing, in the present study, we have applied a simplistic model for Illumina sequences. For instance, the probability of error is uniform along each read, and independent of base, and factors that can cause sequencing bias, like e.g. the read’s GC content [13] or strand [14], are not taken into account. Sometimes a simple model suffices, and it can also make analysis simpler. However, the individual components of FlowSim can easily be replaced by custom tools, and if a more accurate sequencing model is required, it can be implemented separately, and integrated into the simulation pipeline.

Similarly, we could conceive of a more realistic model for the reference genome, in order to explore properties likely to affect our analysis. For instance, repeats caused by recent duplications (common in many plants and teleosts), transposons, or low complexity regions could have dramatic impacts on analysis. Also artifacts of the reference assembly, where chimeric contigs, collapsed repeats, and contamination could have substantial effects on the result. Again, the user is free to implement appropriate designs and insert them as separate stages in the simulation pipeline.

Here we have explored the use of FlowSim for a population genetics study. A similar approach would also allow it to be used for shotgun metagenomics. In that case, the populations would consist of genomes (haplotypes) from different species, instead of originating in a single reference genome. One might also consider mutations of haplotypes in more complex arrangements to emulate evolution of closely related species.

## Availability and requirements

All materials are available on-line, see Table 1 for details. The scripts as well as other tools mentioned are released into the public domain. The documentation for the FlowSim pipeline is available from the Biohaskell Wiki. FlowSim itself is available as Open Source software under the General Public License (GPL) version 2.0.

**Table 1:** On-line resources and supporting materials

FlowSim source	http://malde.org/~ketil/biohaskell/flowsim
code repository	
Documentation	http://biohaskell.org/Applications/FlowSim
Supporting scripts	http://malde.org/~ketil/flowsim-extras

## Competing interests

The author declare that he has no competing interests.
